# Salvage lymph node dissection after ^68^Ga-PSMA or ^18^F-FEC PET/CT for nodal recurrence in prostate cancer patients

**DOI:** 10.18632/oncotarget.21118

**Published:** 2017-09-21

**Authors:** Annika Herlemann, Alexander Kretschmer, Alexander Buchner, Alexander Karl, Stefan Tritschler, Lina El-Malazi, Wolfgang P. Fendler, Vera Wenter, Harun Ilhan, Peter Bartenstein, Christian G. Stief, Christian Gratzke

**Affiliations:** ^1^ Department of Urology, Ludwig-Maximilians-University of Munich, Munich, Germany; ^2^ Comprehensive Cancer Center, Ludwig-Maximilians-University of Munich, Munich, Germany; ^3^ Department of Nuclear Medicine, Ludwig-Maximilians-University of Munich, Munich, Germany

**Keywords:** prostate cancer, radical prostatectomy, biochemical recurrence, salvage lymph node dissection, PSMA PET/CT

## Abstract

The management of patients with biochemical recurrence (BCR) after definitive treatment for prostate cancer remains controversial. Our aim was to determine survival rates and complications of salvage lymph node dissection (sLND) in patients with recurrent prostate cancer after radical prostatectomy, while evaluating biochemical response (BR) with two different positron emission tomography/computed tomography (PET/CT) tracers used for preoperative imaging. sLND was performed in 104 patients diagnosed with isolated nodal recurrence on either ^18^F-fluoroethylcholine (^18^F-FEC) or ^68^Ga-PSMA-HBED-CC (^68^Ga-PSMA) PET/CT. Surgical complications, BR, clinical recurrence (CR), and cancer-specific survival (CSS) were evaluated. Logistic regression was used to determine predictors of complete BR (cBR) and CR after sLND and survival rates were assessed. Median follow-up was 39.5 months. Median patient age and prostate-specific antigen (PSA) at sLND were 64 years and 4.1 ng/mL. Median number of lymph nodes (LNs) removed was 13; median number of positive LNs was 3 per patient. Rate of Clavien-Dindo Grade III complications was low (4.8%). 29.8% of patients developed cBR (PSA < 0.2 ng/mL), and 56.7% partial BR (PSA postoperative < PSA preoperative) after sLND. Patients with LN metastases diagnosed on ^68^Ga-PSMA PET/CT showed a higher rate of cBR compared to ^18^F-FEC PET/CT (45.7 vs. 21.7%, *p* = 0.040). PSA at sLND (*p* = 0.031) and choice of PET tracer (*p* = 0.048) were independent predictors of cBR. The 5-year BCR-free, CR-free and CSS rates were 6.2%, 26.0%, and 82.8%, respectively. While preoperative staging with ^68^Ga-PSMA seems superior, only a limited number of patients developed cBR after surgery. Most patients experienced BCR and CR during follow-up.

## INTRODUCTION

Prostate cancer (PCa) represents the most common noncutaneous malignancy among men [1]. Radical prostatectomy (RP) is one of the definitive treatment options, which may be offered to patients with clinically localized PCa [2]. Despite its curative intent, biochemical recurrence (BCR) due to either local or systemic disease relapse may occur in up to 40–50% of cases after RP at long-term follow-up [3, 4]. According to European Association of Urology (EAU) PCa Guidelines, timing and choice of treatment for BCR without clinical recurrence (CR) after RP are still subject to controversy and may include radiotherapy (RT), intermittent / complete androgen deprivation therapy (ADT), or expectant management [5]. Although only 15% of PCa patients with BCR after RP will die of this disease, approximately one third of these patients will develop CR at follow-up [6].

The site of metastases plays a crucial role in predicting overall survival in this patient cohort [7]. Patients with lymph node (LN) metastases – one of the most common locations of metastatic disease [8] – have a favorable survival outcome compared to patients with bone and/or visceral metastases [7]. Salvage lymph node dissection (sLND) may be offered to patients experiencing isolated nodal recurrence after local treatment [9]. In this patient group, sLND may delay CR and therefore, the use of ADT and its related side effects. Still, there is no proof that sLND significantly prolongs survival. Therefore, it should be considered an experimental approach [9].

Imaging techniques with high sensitivity and specificity are essential for precise preoperative staging. However, preoperative evaluation of nodal involvement even by more advanced imaging modalities such as choline-based positron emission tomography/computed tomography (PET/CT) has demonstrated limited accuracy [10–13], particularly at low prostate-specific antigen (PSA) levels [14]. Prostate-specific membrane antigen (PSMA), a transmembrane protein, is overexpressed on most PCa cells and has been introduced as new target molecule for PCa imaging [15, 16]. The increasing use of ^68^Ga-PSMA-HBED-CC (^68^Ga-PSMA) PET/CT shows promising results in detecting metastatic sites and may overcome this limitation [15, 17–19].

The aim of our study was to evaluate survival rates and peri- and postoperative complications of sLND in PCa patients with isolated nodal recurrence after RP. In addition, we compared biochemical response in patients with two different PET tracers used for preoperative imaging (^18^F-fluoroethylcholine (^18^F-FEC) vs. ^68^Ga-PSMA).

## RESULTS

### Overall patient demographics and clinicopathologic characteristics

Baseline patient demographics and clinicopathologic characteristics at RP and sLND are summarized in Table 1. Overall, 73 patients (71.6%) were classified as high-risk PCa patients (PSA > 20 ng/mL; Gleason score ≥ 8; or ≥ pT3 at RP). After RP, 13 patients (12.6%) underwent RT only, 18 patients (17.5%) underwent ADT only, and 55 patients (53.4%) underwent RT+ADT. Sixteen patients (15.5%) had no further treatment after RP. Prior to sLND, 49 (47.1%), 17 (16.3%), 18 (17.3%), and 20 patients (19.2%) had unilateral pelvic, bilateral pelvic, retroperitoneal and pelvic + retroperitoneal pathologic tracer uptake on PET/CT scan. Median patient age and PSA at sLND were 64 years and 4.1 ng/mL. Site of sLND was restricted to either pelvic (*n* = 50, 48.1%), retroperitoneal (*n* = 7, 6.7%), or pelvic + retroperitoneal (*n* = 47, 45.2%). The median number of removed lymph nodes (LNs) was 13; the median number of positive LNs at histopathology (HP) was 3. Eighty-six patients (82.7%) were found to have HP positive LNs at sLND, of which 51 patients (59.3%) had only positive pelvic LNs, and 35 patients (40.7%) had positive LNs in the retroperitoneum ± pelvis.

**Table 1:** Basic patient demographics and clinicopathologic characteristics at radical prostatectomy and salvage lymph node dissection for all patients and with stratification by PET tracer

Variables	All patients *n* = 104	^18^F-FEC PET/CT *n* = 69	^68^Ga-PSMA PET/CT *n* = 35	*p*-value
PSA at RP, ng/mL				
Median	9.9	9.4	10.9	0.355
IQR	6.5–18.6	6.2–18.4	6.8–20.2	
pT stage at RP, *n* [%]*				
pT2	30 (30.0)	25 (37.9)	5 (14.7)	**0.007**
pT3	65 (65.0)	36 (54.5)	29 (85.3)	
pT4	5 (5.0)	5 (7.6)	0 (0)	
pN stage at RP, *n* [%]*				
pNx	8 (7.8)	5 (7.4)	3 (8.8)	0.284
pN0	59 (57.8)	43 (63.2)	16 (47.1)	
pN1	35 (34.3)	20 (29.4)	15 (44.1)	
Surgical margin at RP, *n* [%]*				
Negative (R0)	54 (55.1)	32 (50.0)	22 (64.7)	0.164
Positive (R1)	44 (44.9)	32 (50.0)	12 (35.3)	
Gleason score at RP, *n* [%]*				
6	5 (5.2)	3 (4.8)	2 (5.9)	0.467
7	40 (41.7)	29 (46.8)	11 (32.4)	
8–10	51 (53.2)	30 (48.4)	21 (61.8)	
No. of high-risk patients, *n* [%]* (PSA > 20 ng/mL; GS ≥ 8; or ≥ pT3 at RP)	73 (71.6)	44 (65.7)	29 (82.9)	0.068
No. of LNs removed at RP				
Median	8	6	10	0.167
IQR	5–12.25	5–12	5–16	
No. of positive LNs at RP				
Median	0	0	0	0.248
IQR	0–1	0–1	0–2	
Time to BCR after RP, months				
Median	26	23	34	0.137
IQR	3.5–46	3–42	4.5–71.25	
Treatment after RP, *n* [%]*				
None	16 (15.5)	5 (7.2)	11 (32.4)	**< 0.001**
RT only	13 (12.6)	6 (8.7)	7 (20.6)	
ADT only	18 (17.5)	16 (15.7)	2 (5.9)	
RT + ADT	55 (53.4)	42 (60.9)	14 (41.2)	
PET/CT positive sites, *n* [%]				
Pelvic unilateral	49 (47.1)	34 (49.3)	15 (42.9)	0.394
Pelvic bilateral	17 (16.3)	9 (13.0)	8 (22.9)	
Retroperitoneal	18 (17.3)	13 (20.3)	4 (11.4)	
Pelvic + retroperitoneal	20 (19.2)	12 (17.4)	8 (22.9)	
Time between RP and sLND, months				
Median	44	44	45	0.889
IQR	21.5–74	23.5–68.5	16–87	
Time between BCR after RP and sLND, months				
Median	7	7	4	0.137
IQR	2–29	2–35	2–11.25	
Age at sLND, years				
Median	64	64	64	0.858
IQR	60–69	60.5–69	59–71	
ASA score at sLND, *n* [%]				
ASA 1	9 (8.7)	7 (10.1)	2 (5.7)	**0.010**
ASA 2	76 (73.1)	55 (79.7)	21 (60.0)	
ASA 3	19 (18.3)	7 (10.1)	12 (34.3)	
PSA at sLND, ng/mL				
Median	4.1	5.9	2.8	**0.021**
IQR	2.0–7.4	2.2–9.8	1.8–5.1	
Site sLND, *n* [%]				
Pelvic	50 (48.1)	27 (39.1)	23 (65.7)	**0.033**
Retroperitoneal	7 (6.7)	6 (8.7)	1 (2.9)	
Pelvic + retroperitoneal	47 (45.2)	36 (52.2)	11 (31.4)	
Total No. of LNs removed				
Median	13	14	10	**0.048**
IQR	7–24.75	9–28.5	4–18	
Patients with HP positive LNs at sLND, *n* [%]	86 (82.7)	56 (81.2)	30 (85.7)	0.562
Site of HP positive LNs at sLND, *n* [%]				
Pelvic only	51 (59.3)	31 (55.4)	20 (66.7)	0.309
Retroperitoneal ± pelvic	35 (40.7)	25 (44.6)	10 (33.3)	
Total No. of positive LNs				
Median	3	3	2	0.514
IQR	1–7	1–7.5	1–5	
Total No. of positive LNs per patient, *n* [%]				
1–2 LNs	33 (38.3)	19 (37.3)	14 (46.7)	0.361
3–5 LNs	23 (26.8)	14 (27.5)	9 (30.0)	
6–10 LNs	15 (17.5)	12 (23.5)	3 (10.0)	
> 10 LNs	15 (17.5)	11 (21.6)	4 (13.3)	
No. of patients with ADT after sLND, *n* [%]*	77 (79.4)	60 (88.2)	17 (58.6)	**0.001**

RP = radical prostatectomy; SD = standard deviation; IQR = interquartile range; PSA = prostate-specific antigen; GS = Gleason score; LNs = lymph nodes; BCR = biochemical recurrence; RT = radiotherapy; ADT = androgen deprivation therapy; PET/CT = positron emission tomography/computed tomography; sLND = salvage lymph node dissection; ASA = American Society of Anaesthesiologists; HP = histopathologically.

*Categories might not total to n due to missing values.

### Patient demographics and clinicopathologic characteristics by PET tracer

Patients who underwent preoperative ^68^Ga-PSMA PET/CT had a lower rate of organ-confined disease at RP (14.7 vs. 37.9%, *p* = 0.007), a lower rate of treatment after RP (67.6 vs. 92.8%, *p* < 0.001), a lower PSA level at sLND (median 2.8 vs. 5.9 ng/mL, *p* = 0.021), a higher rate of pelvic-only sLND (65.7 vs. 39.1%, *p* = 0.033), and less LNs removed at sLND (median 10 vs. 14 LNs, *p* = 0.048; Table 1) compared to patients undergoing ^18^F-FEC PET/CT.

### Postoperative parameters and oncologic follow-up classified by PET tracer

Sixty-nine patients (66.3%) underwent preoperative imaging with ^18^F-FEC PET/CT, 35 patients (33.7%) with ^68^Ga-PSMA PET/CT. Of the entire cohort, 86 patients (82.7%) showed histopathologically (HP) proven LN metastases at sLND (81.2% ^18^F-FEC vs. 85.7% ^68^Ga-PSMA; *p* = 0.562). With regard to PSA response, complete biochemical response (*n* = 31, 29.8%) was significantly higher in patients who had undergone ^68^Ga-PSMA PET/CT (*n* = 16, 45.7%) when compared to the ^18^F-FEC PET/CT group (*n* = 15, 21.7%; *p* = 0.040). Twenty-two patients (71.0%) progressed to BCR after complete biochemical response (93.3% ^18^F-FEC vs. 50.0% ^68^Ga-PSMA; *p* = 0.008). CR occurred in 73 patients (70.2%) with follow-up PET/CT (75.4% ^18^F-FEC vs. 60.0% ^68^Ga-PSMA; *p* = 0.106). As expected, median follow-up after sLND was significantly longer for ^18^F-FEC PET/CT patients (58 months) than for patients undergoing ^68^Ga-PSMA PET/CT (11 months, *p* < 0.001). Of all patients, 20 (19.2%) died of the disease (Tables 1 and 2).

**Table 2:** Postoperative parameters and oncologic follow-up after salvage lymph node dissection classified by PET/CT tracer (^18^F-FEC vs. ^68^Ga-PSMA)

	All patients	^18^F-FEC PET/CT	^68^Ga-PSMA PET/CT	*p*-value
PET tracer, *n* [%]	104/104 (100)	69/104 (66.3)	35/104 (33.7)	
PSA response after sLND, *n* [%]				**0.040**
Complete biochemical response	31/104 (29.8)	15/69 (21.7)	16/35 (45.7)	
Partial biochemical response	59/104 (56.7)	44/69 (63.8)	15/35 (42.9)	
No PSA decrease	14/104 (13.5)	10/69 (14.5)	4/35 (11.4)	
BCR after complete biochemical response, *n* [%]	22/31 (71.0)	14/15 (93.3)	8/16 (50.0)	**0.008**
CR at follow-up, *n* [%]	73/104 (70.2)	52/69 (75.4)	21/35 (60.0)	0.106
Prostatic fossa	11/73 (15.1)	10/52 (19.2)	1/21 (4.8)	
LNs	52/73 (71.2)	35/52 (67.3)	17/21 (81.0)	
Bone	25/73 (34.2)	21/52 (40.4)	4/21 (19.0)	
Visceral	2/73 (2.7)	2/52 (3.8)	0/21 (0)	
Follow-up after sLND, months				**< 0.001**
Median	39.5	58	11	
IQR	21.25–70	39.5–77	8–22	
Cancer-specific mortality at follow-up, *n* [%]	20/104 (19.2)	20/69 (29.0)	0/35 (0)	**< 0.001**

A *p*-value < 0.05 was considered to be statistically significant.

PET/CT = positron emission tomography/computed tomography; LNs = lymph nodes; sLND = salvage lymph node dissection; PSA = prostate-specific antigen; BCR = biochemical recurrence; CR = clinical recurrence; IQR = interquartile range.

### Uni- and multivariate logistic regression analysis predicting complete biochemical response and CR

In univariate logistic regression analysis evaluating pre- and postoperative variates, PSA level and PSA ≤ 4 ng/mL at sLND, and preoperative imaging by ^68^Ga-PSMA PET/CT were significantly associated with complete biochemical response (all *p* < 0.03; Table 3). In multivariate logistic regression analysis, only PSA level at sLND (odds ratio (OR) 0.74; 95% confidence interval (CI) 0.57−0.97, *p* = 0.031), and ^68^Ga-PSMA PET/CT (OR 2.61; 95% CI 1.01−6.76, *p* = 0.048) were independent predictors of complete biochemical response after sLND. Additionally, complete biochemical response was significantly associated with CR after sLND in univariate logistic regression analysis (*p* = 0.026, Table 3).

**Table 3:** Uni- and multivariate logistic regression analysis on clinicopathologic parameters associated with complete biochemical response and clinical recurrence in patients after salvage lymph node dissection

Preoperative variables	Complete biochemical response				Clinical recurrence (CR)
	univariate	multivariate			univariate
	*p*-value	*p*-value	OR	95% CI	*p*-value
PSA at sLND, ng/mL continuous	**0.001**	**0.031**	0.74	0.57–0.97	0.178
PSA at sLND, ng/mL ≤ 4 vs. > 4	**0.021**	0.235	2.57	0.54–12.21	0.145
Gleason score at RP					
6 vs. 7	0.796				0.999
6 vs. 8–10	0.892				0.724
Time, months					
RP → BCR	0.345				0.621
LN status at RP					
pN0 vs. pN1	0.258				0.196
Risk classification					
low-/intermediate-risk vs. high-risk	0.387				0.698
PET/CT tracer					
^18^F-FEC vs. ^68^Ga-PSMA	**0.012**	**0.048**	2.61	1.01–6.76	0.106
Retroperitoneal nodal uptake on preoperative PET/CT	0.168				0.846
Pelvic-only nodal uptake on preoperative PET/CT	0.304				0.884
RT after RP	0.573				0.153
**Postoperative variables**					
Complete biochemical response	-				**0.026**
No. of LNs removed at sLND	0.194				0.957
Positive LNs at sLND					
yes vs. no	0.439				0.719
Site of HP positive LNs at sLND					
pelvic only vs. retroperitoneal ± pelvic	0.059				0.293
No. of positive LNs at sLND	0.100				0.101

A *p*-value < 0.05 was considered to be statistically significant. CR = clinical recurrence; OR = odds ratio; CI = confidence interval; PSA = prostate-specific antigen; sLND = salvage lymph node dissection; RP = radical prostatectomy; BCR = biochemical recurrence; LN(s) = lymph node(s); RT = radiotherapy; PET/CT = positron emission tomography/computed tomography.

### Perioperative parameters and surgical complications associated with sLND

Median duration of sLND was 120 min (including intraoperative frozen section analysis), and median blood loss was 200 mL. Only one patient required a blood transfusion due to hemorrhage. Table 4 depicts peri- and postoperative surgical complications classified by Clavien-Dindo grading system. The most frequent complications included lymphorrhea (*n* = 8, 7.7%) and ileus (*n* = 5, 4.8%).

**Table 4:** Perioperative parameters and surgical complications within 30 days after salvage lymph node dissection classified by Clavien-Dindo

Variables	Values
Duration of sLND*, min	
Median	120
IQR	95–163.25
Blood loss during sLND, mL	
Median	200
IQR	100–300
Blood transfusion during or after sLND, *n* [%]	1 (1.0)
Complications (Clavien-Dindo classification), *n* [%]	
Grade I	
Lymphorrhea	8 (7.7)
Hematoma	2 (1.9)
Grade II	
Ileus	5 (4.8)
Hemorrhage with blood transfusion	1 (1.0)
Deep vein thrombosis	2 (1.9)
Pulmonary embolism	2 (1.9)
Grade IIIa	
Lymphocele requiring drainage	2 (1.9)
Grade IIIb	
Surgical reintervention	3 (2.9)
Wound dehiscence	1 (1.0)
Bladder injury	1 (1.0)
Laparoscopic fenestration of lymphocele	1 (1.0)

sLND = salvage lymph node dissection; IQR = interquartile range.

*Including intraoperative frozen section analysis.

### Survival rates after sLND

**Figure 1:**
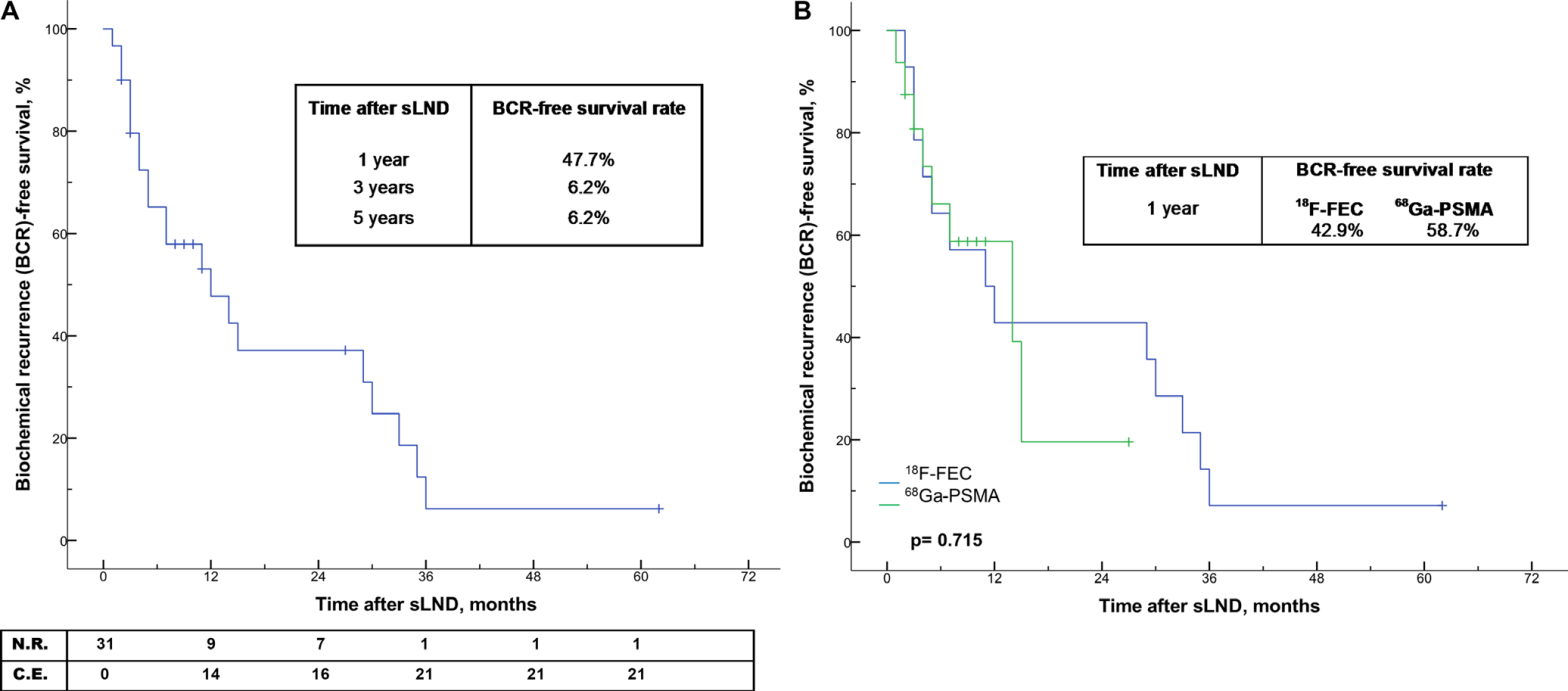
(**A**–**B**) Kaplan-Meier analyses depicting time to biochemical recurrence (BCR) in all patients with complete biochemical response after salvage lymph node dissection (sLND) (*n* = 31; Figure 1A) and with stratification by PET/CT tracer (Figure 1B). Overall median time to BCR after complete biochemical response was 12 months. N.R. = number at risk; C.E. = cumulative events.

**Figure 2:**
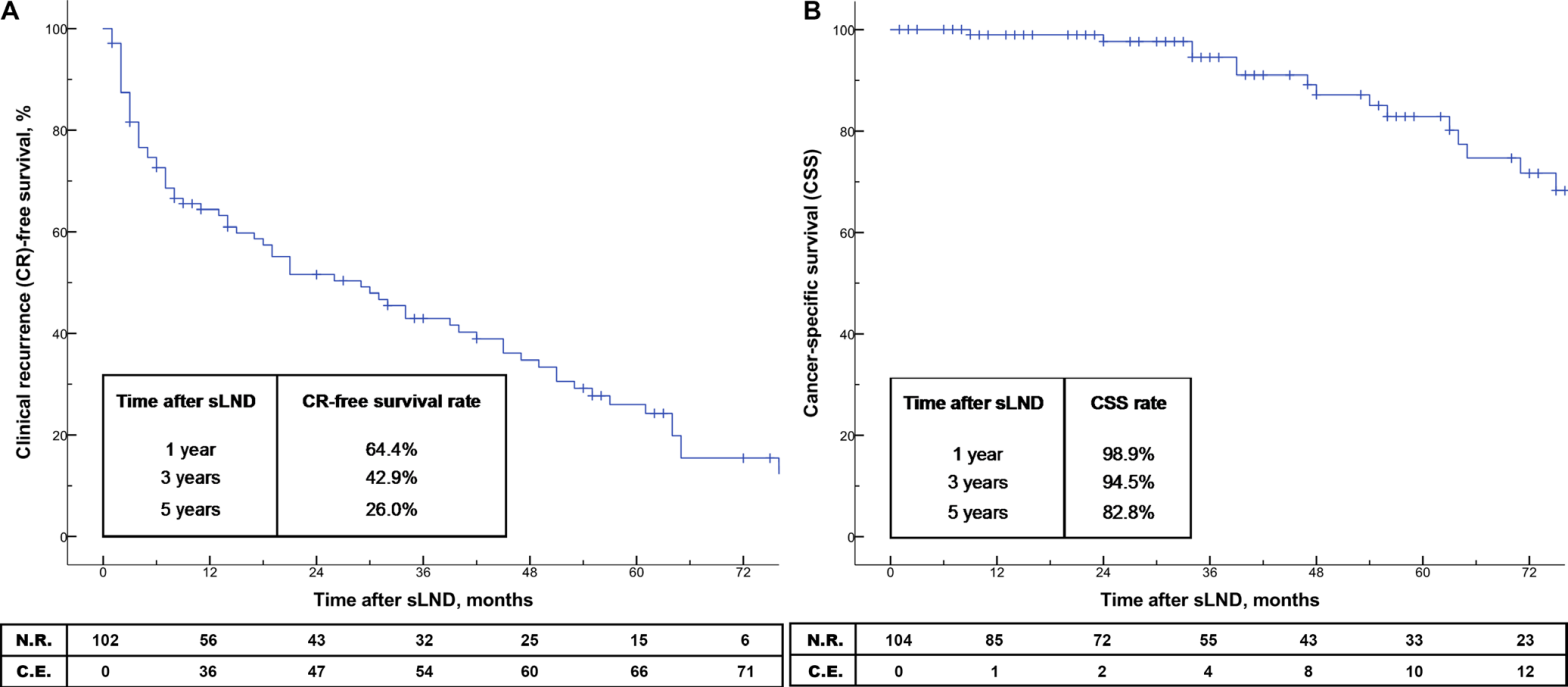
(**A**–**B**) Kaplan-Meier analyses depicting time to clinical recurrence (CR; Figure 2A) and cancer-specific survival (CSS; Figure 2B) after salvage lymph node dissection (sLND) (*n* = 104). Median time to CR and CSS after sLND was 29 months and 104 months, respectively. N.R. = number at risk; C.E. = cumulative events.

**Figure 3:**
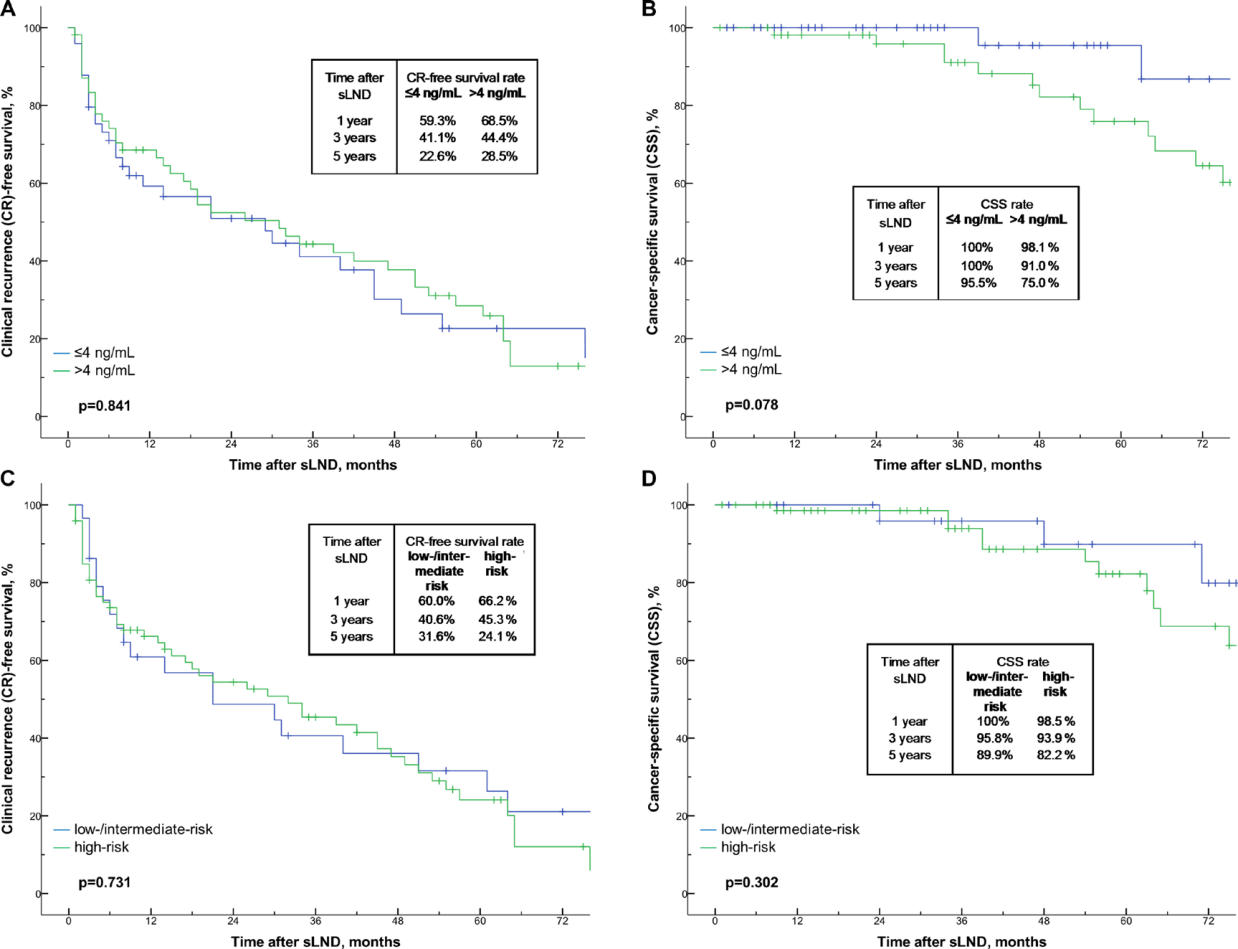
(**A**–**D**) Kaplan-Meier analyses depicting time to clinical recurrence (CR) and cancer-specific survival (CSS) in patients after salvage lymph node dissection (sLND). Patients are stratified by PSA ≤ 4 ng/mL (*n* = 49) and > 4 ng/mL (*n* = 55; Figure 3A–3B) and by risk groups (low-/intermediate-risk *n* = 29 vs. high-risk *n* = 73; Figure 3C–3D).

**Figure 4:**
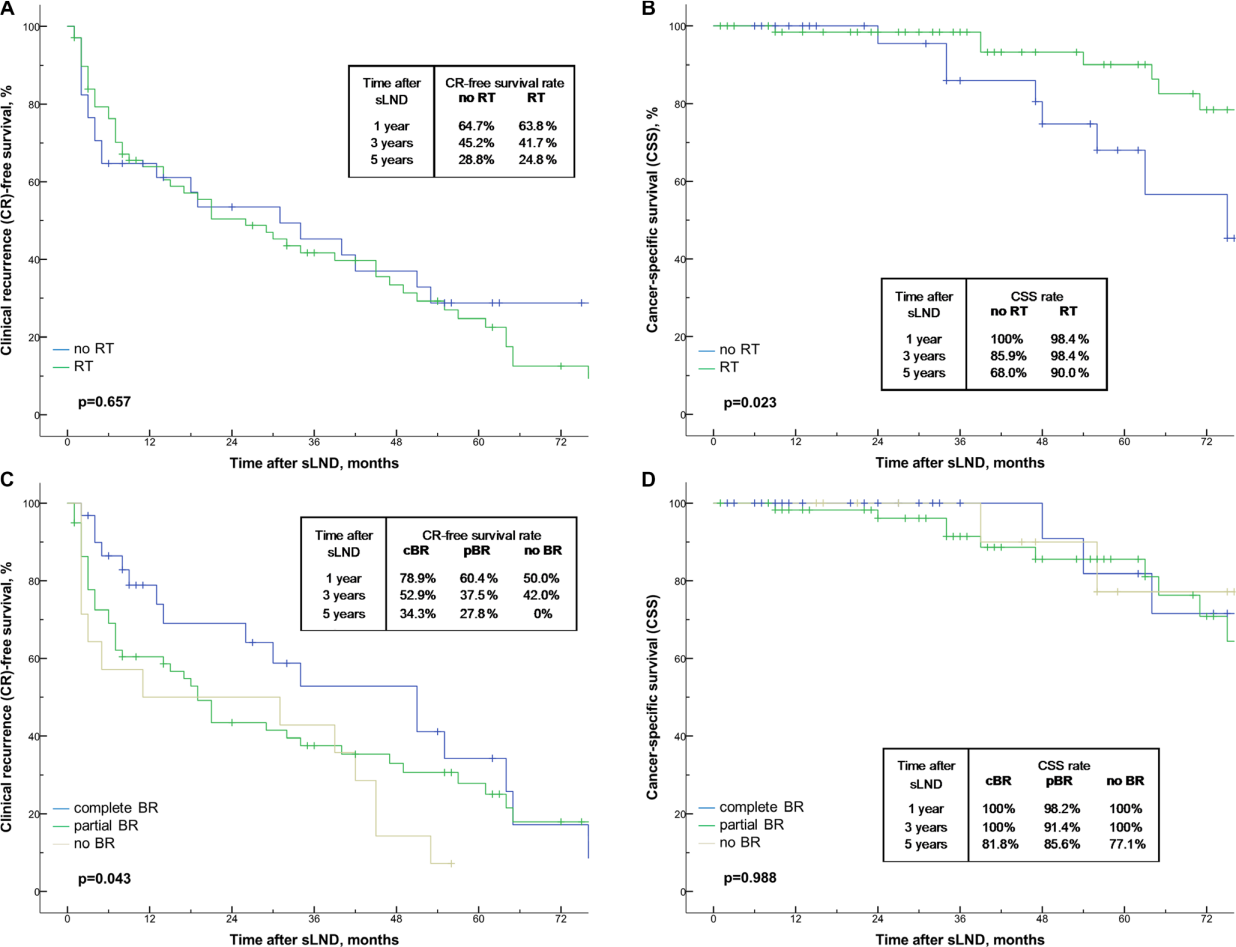
(**A**–**D**) Kaplan-Meier analyses depicting time to clinical recurrence (CR) and cancer-specific survival (CSS) in patients after salvage lymph node dissection (sLND). Patients are stratified by radiotherapy (RT) after radical prostatectomy (RP) (no RT *n* = 34 vs. RT *n* = 68; Figure 4A–4B) and by biochemical response after sLND. Complete (*n* = 31), partial (*n* = 59), and no biochemical response (*n* = 14) are defined as PSA < 0.2 ng/mL, PSA postoperative < PSA preoperative, and no PSA decrease at 40 days after sLND (Figure 4C–4D).

**Figure 5:**
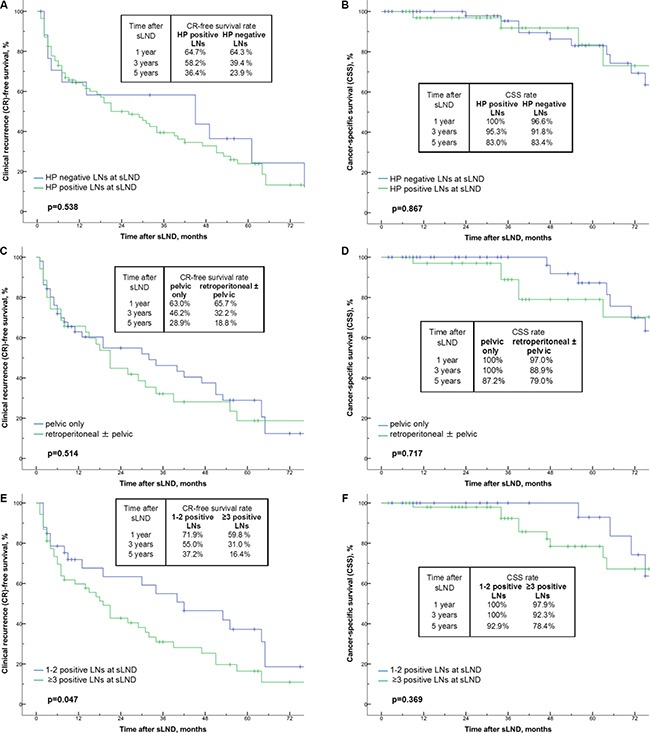
(**A**–**F**) Kaplan-Meier analyses depicting time to clinical recurrence (CR) and cancer-specific survival (CSS) in patients after salvage lymph node dissection (sLND). Patients are stratified by histopathologically (HP) negative (*n* = 18) and positive lymph nodes (LNs) (*n* = 86; Figure 5A–5B), by sites (pelvic only *n* = 51 vs. retroperitoneal ± pelvic *n* = 35; Figure 5C–5D) and by number (1-2 positive LNs *n* = 33 vs. ≥ 3 positive LNs *n* = 53; Figure 5E–5F) of HP positive LNs at sLND.

Of the 31 patients with complete biochemical response after sLND, the 1-year, 3-year and 5-year BCR-free survival rates were 47.7%, 6.2% and 6.2%, respectively (Figure 1A). The median time to BCR was 12 months. When stratifying the patients according to PET tracer, the 1-year BCR-free survival rate were 42.9% for ^18^F-FEC, and 58.7% for ^68^Ga-PSMA without reaching statistical significance (*p* = 0.715; Figure 1B). Overall, the 1-year, 3-year and 5-year CR-free survival and cancer-specific survival (CSS) rates were 64.4%, 42.9%, 26.0% and 98.9%, 94.5%, 82.8%, respectively (Figure 2A–2B). The median time to CR and median CSS were 29 months and 104 months. When patients were stratified according to PSA values (≤ 4 vs. > 4 ng/mL) at sLND and risk groups (low- and intermediate risk vs. high-risk) at RP, CR-free survival and CSS rates did not differ significantly (*p* = 0.841 and *p* = 0.078, and *p* = 0.731 and *p* = 0.302; Figure 3A–3D). However, patients with RT after RP showed significantly better CSS rates (*p* = 0.023; Figure 4A–4B). Similarly, patients with complete biochemical response after sLND demonstrated a significantly improved CR-free survival rate compared to patients with only partial or no biochemical response postoperatively (*p* = 0.043, Figure 4C–4D). Finally, stratification according to HP negative / positive LNs and to sites of positive LNs (pelvic only vs. retroperitoneal ± pelvic) did not show significant changes in survival rates (Figure 5A–5D). Instead, patients with only 1–2 positive LNs at sLND showed a significantly better CR-free survival rate compared to patients with ≥ 3 positive LNs at sLND (*p* = 0.047; Figure 5E–5F).

## DISCUSSION

Despite increased detection and prompt treatment, BCR may occur in a certain amount of patients after definitive treatment for localized PCa [3, 4]. For patients with isolated nodal recurrence, sLND may be considered an individual surgical approach in select patients [9] to potentially delay ADT. However, imaging modalities such as choline-based PET/CT have demonstrated limited accuracy in correctly identifying clinical sites of recurrent PCa [10–12, 15]. The recent utilization of ^68^Ga-PSMA as PET tracer has generated great interest, with the potential to increase detection rates, even at low PSA levels [2, 15, 17]. Eiber et al. found detection rates of 96.8%, 93.0%, 72.7%, and 57.9% for PSA values of ≥ 2, 1 to < 2, 0.5 to < 1, and 0.2 to < 0.5 ng/mL in PCa patients with BCR, respectively [20]. However, previously published studies evaluating oncologic outcomes of patients who underwent sLND for recurrent PCa have used choline-based PET/CT for preoperative localization of nodal recurrence [21–24]. Based upon studies of ^68^Ga-PSMA PET/CT noting improved detection of even small LN metastases (“micrometastases”) [2, 17, 18], one may assume that the oncologic outcome of patients undergoing ^68^Ga-PSMA PET/CT and subsequent sLND for nodal recurrence may also improve due to better identification of positive LNs. In a recently published study from our institution, we demonstrated high concordance rates between ^68^Ga-PSMA PET nodal staging and surgical histopathology after lymph node dissection, both at region level (83%) and patient level (82%) [17]. Several other groups confirmed our findings and reported similarly high detection rates in PCa patients with BCR [19, 20, 25].

In the present study, we analyzed overall survival rates, predictors, and complications of patients undergoing sLND for nodal recurrence. In addition, we aimed to investigate whether the use of two different preoperative PET tracers (^68^Ga-PSMA vs. ^18^F-FEC) leads to a variation in PSA response in these patients. To our best knowledge, we are the first group to highlight short-term oncologic outcome of sLND in patients undergoing preoperative ^68^Ga-PSMA PET/CT.

As shown in Tables 1 and 2, two thirds of patients (66.3%) included in our study underwent preoperative imaging with ^18^F-FEC PET/CT and one third underwent imaging with ^68^Ga-PSMA PET/CT. This unequal distribution is attributable to ^68^Ga-PSMA PET/CT replacing ^18^F-FEC PET/CT as the imaging modality for prostate cancer staging in our department from November 2013 onwards. When comparing the two cohorts, patients who underwent preoperative ^68^Ga-PSMA PET/CT were noted to have significantly lower PSA levels at sLND, a higher rate of pelvic-only sLND, and less LNs removed at sLND compared to patients undergoing ^18^F-FEC PET/CT. Interestingly, a significant difference in PSA response after sLND could be observed between the two groups. In the ^68^Ga-PSMA PET/CT cohort, almost half of the patients achieved complete biochemical response (PSA < 0.2 ng/mL at 40 days after sLND), whereas the rate of complete biochemical response in patients with preoperative ^18^F-FEC PET/CT was significantly lower (45.7% vs. 21.7%, *p* = 0.040). The significant increase of complete biochemical response in patients with preoperative ^68^Ga-PSMA PET/CT is supported by several studies showing an increased detection rate by ^68^Ga-PSMA PET compared to other imaging modalities - even at lower PSA levels, as mentioned above [10, 17, 25, 26]. Therefore, a more targeted sLND approach with resection of less LNs based upon ^68^Ga-PSMA PET/CT findings appears to be justified, and is supported by improved complete biochemical response in our study cohort. However, future studies need to compare region-based vs. extended sLND as well as unilateral vs. bilateral sLND with regard to oncologic outcome.

Additionally, a higher rate of 1-year BCR-free survival after complete biochemical response was noted in the ^68^Ga-PSMA PET/CT group when compared to the ^18^F-FEC PET/CT group (Figure 1B). However, this finding may have resulted from the significant difference in length of follow-up between the two groups (median 58 vs. 11 months, *p <* 0.001) and potential bias attributable to the fact that ^68^Ga-PSMA has only been introduced a few years ago [16]. In order to validate these preliminary observations, further long-term follow-up is required for patients undergoing ^68^Ga-PSMA PET/CT before sLND.

When comparing our findings to those of other sLND series, our overall complete biochemical response rate after sLND (29.8%), BCR-free and CR-free survival rates were lower than other previously published sLND series [22, 23, 27]. Only the study of Jilg et al., demonstrating a 5-year BCR-free survival rate of 8.7% and 5-year CR-free survival rate of 25.6%, showed similar results [24]. This is of particular interest since Jilg and colleagues performed bilateral sLND even for unilateral positive findings only. Mean PSA at sLND (11.1 ng/mL), rates of ADT prior to sLND (78.7%) and pelvic-only sLND (54.0%) were also higher in this study [24], potentially explaining the differences in oncologic outcome observed in previous analyses [22, 23, 27, 28]. Moreover, we included a higher proportion of patients with advanced, high-risk disease (71.6%) in our analysis (Table 1), which might have influenced oncologic outcome [22–24, 27, 28].

Compared to previous studies, we found similar independent predictors associated with improved survival rates after sLND [21–24, 27]. Patients with ≤ 2 positive lymph nodes at sLND and complete biochemical response after sLND had significantly better CR-free survival rates at follow-up (Figure 5E). For CSS, patients with RT after RP showed significantly better survival rates (Figure 4B). In our analysis, PSA values < 4 vs. ≥ 4 ng/mL, prostate cancer risk stratification at RP (Figure 3A–3D), or histopathology findings at sLND (Figure 5A–5D) did not significantly influence CR-free survival and CSS rates. However, PSA level at sLND and preoperative imaging with ^68^Ga-PSMA PET/CT were independent predictors for complete biochemical response at multivariate logistic regression analysis (Table 3).

In our study population, the overall number of surgical complications within 30 days after sLND was low (Table 4). The most frequent complications were mild according to Clavien-Dindo classification and included lymphorrhea and ileus (Grade I and II). Surgical reintervention was only required in 3 patients (Grade IIIb), and blood transfusion due to hemorrhage in 1 case (Grade II). Our data is in concordance with previously published studies demonstrating that sLND is a feasible, safe approach with no reported postoperative mortality to date [23, 24, 27].

Despite several strengths, our study has inherent limitations. First, the retrospective design of the study and the lack of a control group treated with ADT prevent comparison of survival rates between sLND and the standard of care; future randomized controlled studies are needed. Broad inclusion criteria, different group sizes and various patient characteristics in both PET groups may have led to a rather high degree of patient heterogeneity and selection bias. In particular, there was no restriction regarding RT after RP or ADT before and after sLND. The relatively low number of removed LNs per patient and the high percentage of patients who received ADT after sLND might also introduce bias. Additionally, patients with lower tumor burden might have preferably selected for sLND. Although we provided a mean follow-up of almost four years, the significant difference in length of follow-up between the two PET/CT cohorts may introduce lead-time bias when comparing overall survival rates.

However, despite these limitations, our study is the first to compare rates of short-term biochemical response after sLND in patients undergoing either ^18^F-FEC PET/CT or ^68^Ga-PSMA PET/CT as preoperative imaging modalities, hereby adding new knowledge to existent sLND data. Rather than an extended sLND on patients with isolated nodal recurrence, targeted lymph node dissection based upon ^68^Ga-PSMA PET/CT findings may be feasible due its higher level of accuracy compared to choline-based PET/CT [25, 26]. PSMA-radioguided surgery using a probe intraoperatively may also facilitate sLND [29]. However, in the current clinical setting sLND based on ^68^Ga-PSMA PET/CT findings may represent a more suitable, less time consuming approach, even for non-tertiary care centers [17].

In conclusion, we could demonstrate that sLND is feasible and may be safely performed in patients experiencing isolated nodal recurrence after RP. However, complete biochemical response after surgery can only be achieved in a subset of patients. PSA level at sLND and preoperative imaging with ^68^Ga-PSMA PET/CT appear to be independent predictors of complete biochemical response. The majority of patients will progress to BCR and CR during follow-up. Proper patient selection seems essential for this individual surgical approach. Thus, future prospective randomized trials with long-term follow-up are needed in order to seek further evidence for the potential survival benefit of sLND.

## MATERIALS AND METHODS

### Patient identification

A total of 104 consecutive patients with BCR after RP for PCa were identified. In accordance to international guidelines, BCR was defined as two consecutive PSA rises > 0.2 ng/mL after RP. All patients had increased tracer uptake in at least 1 LN on either ^18^F-FEC or ^68^Ga-PSMA PET/CT indicating the presence of LN metastases. Patients with evidence of local recurrence, bone or visceral metastases on PET/CT were excluded from the analysis. All patients underwent sLND at our urology department from June 2005 to July 2016. Data were prospectively collected in our clinical database. All patients signed written informed consent before surgery highlighting the experimental character of this surgical approach.

### PET/CT imaging

Tracer application, PET/CT scanning procedure and subsequent image analysis were performed as described in detail previously [10, 17, 30]. Patients were administered intravenously either ^18^F-FEC (until October 2013) or ^68^Ga-PSMA (from November 2013 onwards).

### sLND and histopathologic evaluation

An open approach through an abdominal midline incision was used. sLND after preoperative ^18^F-FEC PET/CT was performed as described by our group previously [21]. For the ^68^Ga-PSMA PET/CT cohort, we performed sLND based on specific regions according to the most recent PET/CT findings. All dissected LNs were classified according to their anatomic region and immediately sent for histopathologic analysis. LNs were evaluated according to standard protocols with serial sectioning (200 μm slices) by standard hematoxylin and eosin (H&E) staining. All LNs negative by H&E underwent further evaluation by immunohistochemistry for cytokeratins and PSA to rule out micrometastases. Histopathologic evaluation was performed by a highly experienced, designated uro-pathologist. Surgical complications were documented and classified using the Clavien-Dindo grading system [31].

### Patient follow-up

Follow-up PSA testing was performed 40 days postoperatively, and every 3 to 6 months thereafter. Postoperative PET/CT scan was performed according to persistently elevated and/or rising PSA, patient's clinical symptoms, and/or patient preference. Additional treatment after sLND such as ADT (luteinizing hormone-releasing hormone agonists or antagonists ± anti-androgens) or RT was recommended depending on PSA levels, patient's clinical symptoms, or PET/CT results at follow-up.

### Oncologic outcomes

Biochemical response, BCR after biochemical response, CR after sLND, and CSS after sLND were used as oncologic outcome variables. Complete biochemical response was defined as PSA < 0.2 ng/mL at first evaluation 40 days after sLND, and partial biochemical response as postoperative PSA less than preoperative PSA. Two consecutive PSA rises > 0.2 ng/mL determined BCR after sLND. CR was detected by positive PET/CT scan demonstrating new lesions (prostatic fossa; LN, bone or visceral metastases) after sLND in the presence of rising PSA. Time and cause of death were evaluated by chart review, death certificates, or treating physicians.

### Statistical analysis

Continuous variables were presented as the median (interquartile range, IQR). Categorical variables were reported using n and frequencies. Continuous and categorical variables were compared between groups with Mann-Whitney *U* test and chi-square test, respectively. Kaplan-Meier curves and the log rank test were used to evaluate BCR in patients with complete biochemical response, time to CR and CSS. Uni- and multivariate logistic regression models were used to identify potential predictors of complete biochemical response and CR. A *p*-value < 0.05 was considered to be statistically significant. All calculations were performed using SPSS Statistics software, version 24.0 (IBM, Armonk, NY, USA) and STATISTICA 13 (Dell Statistica, Tulsa, OK, USA).

## Abbreviations

^18^F-FEC: ^18^F-fluoroethylcholine
^68^Ga-PSMA: ^68^Ga-PSMA-HBED-CC
ADT: androgen deprivation therapy
BCR: biochemical recurrence
BR: biochemical response
C.E.: cumulative events
cBR: complete biochemical response
CI: confidence interval
CR: clinical recurrence
CSS: cancer-specific survival
GS: Gleason score
HP: histopathology, histopathologically
IQR: interquartile range
LN(s): lymph node(s)
N.R.: number at risk
OR: odds ratio
PCa: prostate cancer
PET/CT: positron emission tomography/computed tomography
PSA: prostate-specific antigen
PSMA: prostate-specific membrane antigen
RP: radical prostatectomy
RT: radiotherapy
sLND: salvage lymph node dissection
